# Forest Restoration and Parasitoid Wasp Communities in Montane Hawai’i

**DOI:** 10.1371/journal.pone.0059356

**Published:** 2013-03-19

**Authors:** Rachelle K. Gould, Liba Pejchar, Sara G. Bothwell, Berry Brosi, Stacie Wolny, Chase D. Mendenhall, Gretchen Daily

**Affiliations:** 1 Emmett Interdisciplinary Program in Environment and Resources, Stanford University, Stanford, California, United States of America; 2 Center for Conservation Biology, Stanford University, Stanford, California, United States of America; 3 Department of Fish, Wildlife, and Conservation Biology, Colorado State University, Fort Collins, Colorado, United States of America; 4 Department of Environmental Studies, University of California Santa Cruz, Santa Cruz, California, United States of America; 5 Department of Environmental Studies, Emory University, Atlanta, Georgia, United States of America; 6 Natural Capital Project, Stanford University, Stanford, California, United States of America; 7 Department of Biology, Stanford University, Stanford, California, United States of America; Lakehead University, Canada

## Abstract

Globally, most restoration efforts focus on re-creating the physical structure (flora or physical features) of a target ecosystem with the assumption that other ecosystem components will follow. Here we investigate that assumption by documenting biogeographical patterns in an important invertebrate taxon, the parasitoid wasp family Ichneumonidae, in a recently reforested Hawaiian landscape. Specifically, we test the influence of (1) planting configurations (corridors versus patches), (2) vegetation age, (3) distance from mature native forest, (4) surrounding tree cover, and (5) plant community composition on ichneumonid richness, abundance, and composition. We sampled over 7,000 wasps, 96.5% of which were not native to Hawai’i. We found greater relative richness and abundance of ichneumonids, and substantially different communities, in restored areas compared to mature forest and abandoned pasturelands. Non-native ichneumonids drive these differences; restored areas and native forest did not differ in native ichneumonid abundance. Among restored areas, ichneumonid communities did not differ by planting age or configuration. As tree cover increased within 120 m of a sampling point, ichneumonid community composition increasingly resembled that found in native forest. Similarly, native ichneumonid abundance increased with proximity to native forest. Our results suggest that restoration plantings, if situated near target forest ecosystems and in areas with higher local tree cover, can facilitate restoration of native fauna even in a highly invaded system.

## Introduction

Ecological restoration efforts to conserve both biodiversity and ecosystem services are increasingly common [1]. The success of restoration remains poorly known, however, because of tendencies to monitor only a few ecosystem components [2] and for only a few years after restoration activity [3]. The majority of projects are evaluated on progress towards restoring physical structure (flora or physical features) or vertebrate species of concern, while rarely measuring effects on other taxa or ecosystem processes [4], [5]. Among more systematically monitored projects, many fail to achieve the targeted ecosystem’s species composition, structure, or function [6], [7].

Arthropods play important roles in ecosystems as pollinators, predators, parasites, and prey [8]. Their small size, short life cycles, and large numbers facilitate use as indicators of overall biodiversity and ecosystem stability [9], [10]. The use of arthropods to monitor restoration progress, however, also has a notable disadvantage: the scarcity of life history and ecological data on most arthropod species make some ecological analyses difficult [9], [11]. Among studies of arthropod response to restoration of native plants, some report successful arthropod community restoration compared to the reference ecosystem in the long term (30 years) [11], and even in the short term (<6 years [8], [12], [13]). The definition of “success” varies [14], [15]. One of these studies, for example, found common arthropods in similar densities on planted and naturally occurring shrubs in California scrubland, but that planted shrubs were less likely to support rare species [13]. Other studies have found markedly dissimilar communities between restored and reference sites in both the short and long term [9], [11], [16]; one documented greater butterfly richness and abundance in restored areas than in control areas [17].

Driven by mixed results from past analyses of arthropods in restoration, we explore the effect of efforts to restore pasturelands to montane Hawaiian forest on the parasitoid wasp family Ichneumonidae. This family, with ca. 60,000 species [18], is one of the most biologically diverse insect families in the world. We chose to focus on ichneumonids for a number of reasons. First, the native Hawaiian ichneumonid fauna includes at least three subfamilies and 38 species [19]. Second, Ichneumonids are relatively host-specific parasitoids whose diversity is thought to reflect that of their hosts [20] and sometimes other arthropod groups [21]; relatedly, their complex trophic roles can lead to substantial impacts on other species when icheumonids are introduced into new ecosystems [22]. Third, they appear to disperse readily to suitable habitat [23] and to indicate environmental change [24].

Another important reason for selecting this taxon is that many native and non-native ichneumonids in Hawai’i parasitize Lepidoptera [19]. Biologists working in Hawai’i have suggested that non-native dominance of ichneumonid communities may impact threatened native birds by depleting their prey through parasitism [25]. The fairly well-known dominance of Hawaii’s ichneumonid fauna by non-natives [19], [26] does not detract from the taxon’s relevance to restoration due to the extraordinary role played in Hawai’i by non-native species [27], including flora [28], arthropods [27], and birds [29]. Hawai’i provides a rich example of a system for which restoration cannot proceed without accounting for non-native species. We thus seek to understand the impacts of restoration on a biologically informative taxon despite the high proportion of non-native species in its Hawaiian populations.

In this study we explore whether planting of a dominant native tree species (*Acacia koa*), along with a number of native understory trees and shrubs, yields an ichneumonid wasp fauna resembling communities found in nearby native Hawaiian forest. We investigated an ongoing, large-scale restoration effort in the Hakalau Forest National Wildlife Refuge in Hawai’i, focusing on distance to native forest, planting configuration, and time since planting. We frame our habitat restoration study around both native and non-native species to address the following questions: 1) Do Ichneumonidae use reforested *A. koa* stands?; 2) How do ichneumonid communities in restored habitats compare with those in native forest, the target ecosystem?; and 3) Does ichneumonid community composition, particularly with respect to native species, vary as a function of a) age of planting, b) planting configuration (patches versus corridors), c) distance from native forest, d) surrounding tree cover, or e) components of the understory plant community?

## Materials and Methods

### Ethics Statement

We received permission to conduct this study and to collect insect samples in the form of Special Use Permits issued by the U.S. Fish and Wildlife Service in 2007 (SUP# 12516-07018) and 2008 (SUP#12516-08022). No protected species were sampled.

### Study Site

**Figure 1:**
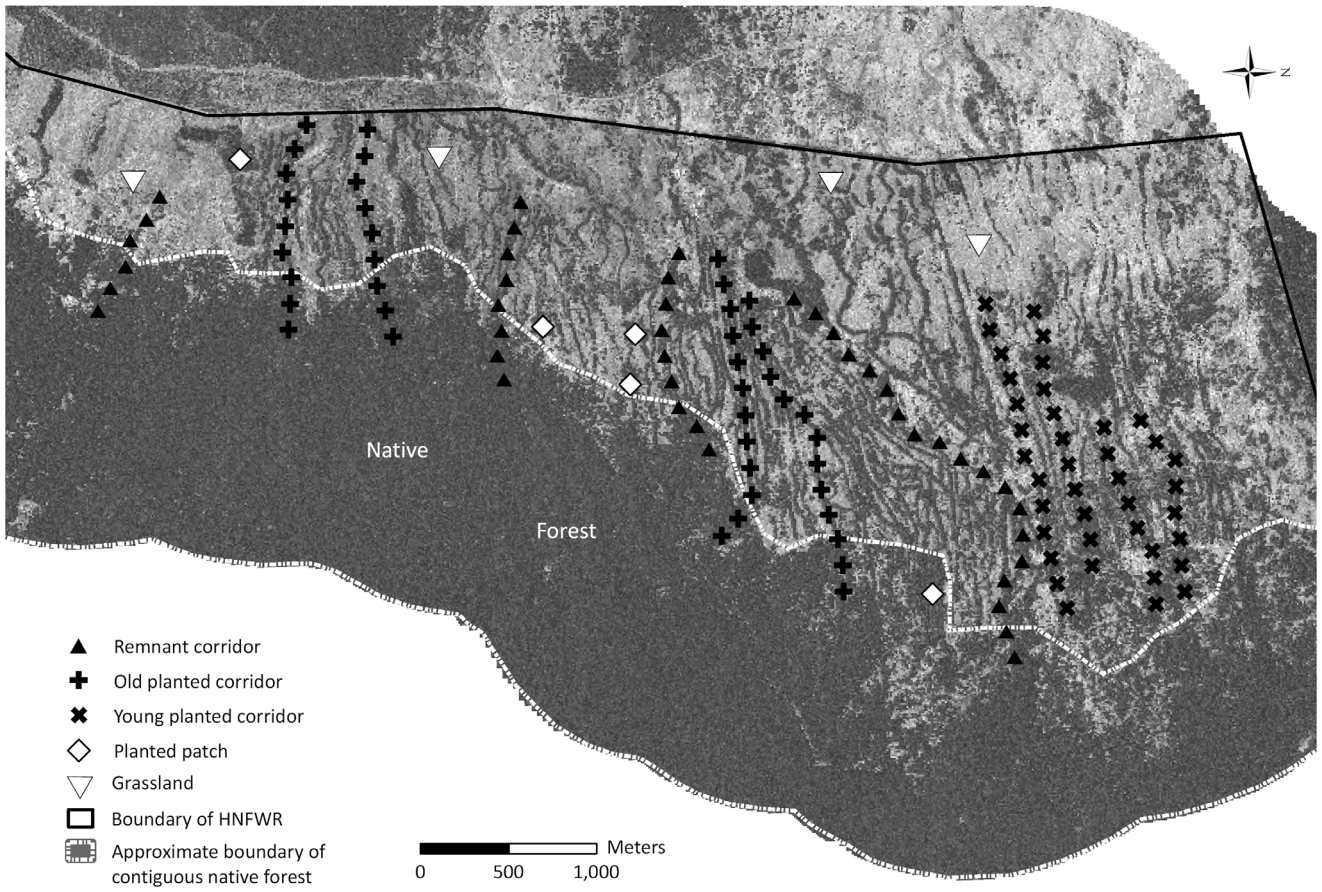
Map of study site, Hakalau National Forest Wildlife Refuge. Geometric figures indicate sampling points and corresponding habitat types. Sampling points and a designation of the approximate boundary of the native forest (dotted white line) are overlaid on a 0.5 m-resolution aerial photograph showing tree cover (dark areas are the forest, corridors, and patches).

We sampled ichneumonids in the Hakalau Forest National Wildlife Refuge (HFNWR), a United States government-owned 10,500 ha reserve on the eastern slope of Hawai’i Island (Fig. 1). Native, mature forest covers ca. 7,000 ha of the reserve (the portion below ca. 1,750–1,900 m elevation). Dominant canopy species in this forest are *Acacia koa* (koa) and *Metrosideros polymorpha* (‘ōhi’a); the understory is comprised mostly of native trees and ferns, with some non-native trees, forbs, and lianas. The remaining 3,500 ha at higher elevations, although originally forested, were cleared for cattle pasture in the early to mid-1800’s and are now dominated by non-native grasses. The U.S. Fish and Wildlife Service acquired this land in 1986, primarily to protect and restore prime habitat for Hawaii’s highly endangered forest bird species. The HFNWR initiated reforestation in former pastureland in 1987 [30], 20 years before we conducted this study, and reforestation efforts continue today. We sampled within stands that were planted from the late 1980s through the early 2000s and were 5–20 years old at the time of this study.

### Habitat Types

HFNWR’s reforestation program involved planting primarily koa seedlings. Koa was selected for its rapid growth and ability to survive in high-light, non-forest conditions at high elevations. Planting was conducted in two designs: linear corridors stretching uphill from within the mature forest, and roughly rectangular patches of trees in the middle of pasture. Planting locations were selected without reference to soils or geomorphology (see Fig. 1). Since 2003 there has been regular planting of native understory trees and shrubs within these koa corridors and patches. At the time of this study, natural regeneration under planted koa was limited to several fern species (personal observations) and the shrub *Vaccinium reticulatum*. Corridors are approximately 40 m wide and range from 0.5 to 2.5 km in length. Patches are roughly rectangular, with sides of 70–125 m.

The landscape now comprises a variety of different habitat types, referenced here as:

1. *Native forest* – contiguous native forest; the target system of the restoration effort.
2. *Remnant corridors* – corridors of mature (unplanted), native trees persisting in former pasturelands in steep gulches, somewhat protected from former cattle disturbance.
3. *Old planted corridors* – corridors planted with koa 11–20 years before this study; these corridors were planted on relatively flat land.
4. *Young planted corridors* – corridors planted with koa 5–10 years before this study; these corridors were planted on relatively flat land.
5. *Patches* – stands of koa planted 5–20 years before this study.
6. *Grassland* – former pastureland, dominated by non-native grasses.

### Ichneumonid and Vegetation Sampling

We sampled plants and arthropods at HFNWR from June–August 2007 and June–August 2008. We surveyed 12 corridors (four remnant corridors, four young planted corridors, and four old planted corridors), five patches of restored koa, and four grassland sites. We identified suitable replicate corridors and stands using aerial images, historical records, and expert field-based knowledge. The entire study site (Fig. 1) extends about 6 km north-south, and 3 km east-west. All sampling points are between 1,650 and 2,000 m elevation.

For each corridor, we surveyed fixed points starting 300 m within the native forest adjacent to the corridor and continuing at 150 m intervals along each corridor’s length. The first points, at 300 m and 150 m inside the native forest, constitute our native forest sampling (Fig. 1). At each patch and grassland site, our goal was to collect a representative sample of wasps from the area. In patches, we determined center points using aerial photographs in a Geographic Information System; we measured the length and width of patches, identified the center point, and subsequently found these points in the field using UTM coordinates. We selected sites for grassland sampling by using these same tools to identify the points on the Refuge furthest from the nearest tree cover. We established survey locations at the patch center or grassland site ‘center’ and 50 m from those points at 0°, 120°, and 240°. We combined data from all four sampling locations in each patch and grassland; we situated survey points in three different directions 50 m away simply as a non-biased method for increasing sampling coverage.

#### Ichneumonid sampling

We surveyed arthropods using six pan traps at each point. Pan traps were 20-cm-diameter plastic bowls [31], placed on the ground 1–2 m apart in the vicinity of each sampling point. We used three colors (two blue, two yellow, and two white at each point) to attract a diversity of invertebrates, since species may respond differently to the color spectrum. We filled traps approximately 2/3 full of water; to decrease surface tension we added biodegradable soap (1 ml per liter of water). We left the traps out 24 hours (12 hours of daylight) unless weather conditions during part or all of that time were such that wasps were unlikely to be active (e.g., high humidity or high wind conditions [32]). In those cases we left the traps out longer in order to ensure 12 hours of “effective” trapping time. We combined samples from all six traps at each point. We repeated this survey twice in Summer 2007 and three times in Summer 2008, for a total of five summer sampling days at each point.

We built a reference collection of Ichneumonidae and identified them to subfamily using Goulet and Huber (1993) and then to genus or species using the “Key to the Ichneumonids of Hawaii” [33]. For one species not in the key, we consulted an expert [34]. We followed the native/non-native descriptions of Hawaii’s Bishop Museum [35]. Our data are publicly available in the Dryad database at www.datadryad.org, http://dx.doi.org/10.5061/dryad.dr6s2.

#### Vegetation sampling

In all but remnant corridors, we recorded the abundance and size class of all vegetation in a 10×10 m^2^ square centered on the sampling point. In the excessively steep terrain of remnant corridors, which typically had woody vegetation that extended only 5 m away from a deep gulch, we sampled vegetation in a 5×10 m^2^ rectangle centered on the sampling point.

### Data Analysis

We tested the effects of habitat type, surrounding tree cover, and distance to forest on ichneumonids using a number of analyses. First, we compared ichneumonid richness and abundance across habitat types (section 2.4.1). Second, we analyzed ichneumonid community composition variation across habitat types (section 2.4.1). Third, we examined whether three response variables [(a) ichneumonid species richness, (b) native ichneumonid abundance, and (c) the similarity of individual sites to the pooled community of ichneumonids in forest habitats] varied with respect to three explanatory variables [(a) distance to forest, (b) tree cover, and (c) for abundance of native ichneumonids only, the non-koa plant community] (section 2.4.2).

We do not consider samples within the same corridor, patch, or grassland independent from one another; nor did we consider the temporal replicates from each point independent. We minimize the risk of pseudoreplication using the techniques described below and detailed in the Supporting Information (S1. Elaboration of Methods).

#### Comparison of wasp communities across the landscape

To assess whether ichneumonids visit restoration plantings differently than they do adjacent unrestored areas, we calculated sampled species richness and individual abundance. To determine statistical significance of observed differences, we used a Generalized Linear Mixed Model, a method described in more detail below and in the Supporting Information (S1).

To compare wasp communities across habitats, we used permutational, nonparametric multivariate analysis of variance tests (“PerMANOVA”) [36]. We conducted one global analysis, and then pairwise analyses for habitat pairs of interest. We conducted PerMANOVA analyses using the “adonis” function in R [37]. To prevent pseudoreplication in the PerMANOVA tests, we pooled temporal replicates and used only one sampling point from each corridor, patch, and grassland. The Supporting Information (S1) details the points used in each habitat type and two alternative verifications of the results. We conducted non-metric multidimensional scaling to aid in visualization of relationships presented below.

We based community similarity tests on Chao similarity coefficients [38] to account for our many rarely encountered species (Table 1). We also developed a similarity-to-forest index based on Chao similarity coefficients; the index quantifies community composition similarity of each sampling point in the corridors with a set of sampling locations in the target ecosystem (12 points, each 300 m inside the forest edge; see Figure 1). We calculated this index for each point by calculating Chao similarity coefficients (denoted as *C*) for each planted point (denoted as *k*) compared with each forest point (denoted as *f_i_*) (see Eq. 1). We then calculated the arithmetic mean of those 12 Chao similarity coefficients for each planted point (denoted as *Mp*):
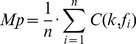
(1)


**Table 1:** The scientific name, status (native or introduced) and abundance of all ichneumonids sampled in this study.

Species	Native or introduced?	Abundance
Agasthenes swezyzi	Introduced	3127
Barichneumon californicum	Introduced	7
Diadegma blackburni	Introduced	336
Diadegma insularis	Introduced	53
Diplazon laetatorius	Introduced	23
Enicospilus sp.1	Native	2
Gelis sp.1	Introduced	60
Gelis tenellus	Introduced	8
Hyposoter exiguae	Introduced	213
Ichneumon cupitus	Introduced	204
Ichnuemon purpuripennis	Introduced	74
Ichnuemon lugubrator	Introduced	2
Ichnuemon near laetus	Introduced	31
Ichnuemon sp.1 (near lugubrator)	Introduced	99
Megastylus flavopictus	Introduced	2
Pimpla punicipes	Introduced	395
Pristomerus hawaiiensis	Native	6
Rubicundiella perturbatrix	Introduced	56
Spolas sp.1	Native	265
Vulgichneumon diminutus	Introduced	1381
Woldstedtius flavolineatus	Introduced	1380

To convert the results into a more easily interpretable 0–1 format, we standardized values by dividing each *Mp* value by the maximum *Mp* value in the dataset (*Mp_max_*):
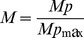
(2)


Because the similarity-to-forest index was created from proportional data, we transformed the data using the logit transform so that they closely fit normality assumptions [39].

#### Effects of distance to forest, tree cover, and plant community

We used Generalized Linear Mixed-Effects Models (GLMM) [40] to quantify the impact of three explanatory variables [distance from forest, tree cover, and presence/absence of non-koa plants (Table 2 and Table S1)] on three response variables [ichneumonid species richness, similarity-to-forest index, and abundance of native Ichneumonids]. All response variables exhibited spatial autocorrelation as measured by Moran’s I (Appendix A). The GLMM addressed this autocorrelation by grouping spatially proximate observations (through the specification of corridor as a random effect); sampling points within corridors were not considered independent from one another (spatially or otherwise). Similarly, when all temporal replicates were used in analyses, the GLMM addressed the non-independence of data from the same sampling point (through the specification of sampling point as a random effect). That is, the GLMM approach avoided pseudoreplication by accounting for the non-independence of sampling points while using all spatial and temporal replicates [41], [42].

**Table 2:** Summary of GLMM Analyses.

Question	Response Variable(s)	Explanatory Variable(s)	Random Effects Structure	Error Structure	Notes
Do richness andabundance of ichneumonidsvary between pastureandrestoration sites?	Ichneumonid richnessIchneumonid abundance	Habitat type (grassland vs. planted sites)	Point nested within corridor	Poisson	(1) Forest and remnant corridors excluded (interest was in whether ichneumonids were found in restoration plantings)
Is the similarity-to-forest index correlated with distance to forest or tree cover?	Similarity-to-forest index	Distance to forestTree cover	Point nested within corridor	Normal (Gaussian)	(1) Only forest and corridor points used (distance gradient only present in corridors) (2) All points 300 m into forest omitted (because involved in similarity-to-forest calculation) (3) Points >1.5 km from forest omitted (because at distances >1.5 km sample sizes are too low)
Is native ichneumonid abundance correlatedwith distance fromforest or tree cover?	Abundance of all three native ichneumonidspecies found	Distance from forestTree cover	Point nested within corridor	Poisson	(1) Only forest and corridor points used (distance gradient only present in corridors) (2) Points >1.5 km from forest omitted (because at distances >1.5 km sample sizes are too low).
Does native ichneumonid presence differ by habitat type?	Abundance of all three native ichneumonidspecies found	Habitat type	Point nested within corridor	Poisson	
Is the presence of *Spolas* sp.1 correlated with plant community composition?	Presence/absence of*Spolas* sp.1	Presence/absence of plant species	Corridor	Binomial	(1) Only *Spolas* sp.1 used in this analysis, due to the host-specific nature of plant-invertebrate host-parasitoid interactions (2) Temporal replicates pooled

We specified a Poisson error structure for count data, a binomial error structure for presence/absence data, and a Gaussian error structure for proportional data. All count and presence/absence data were neither overdispersed nor underdispersed, and proportional data fit normality assumptions after logit transformation [39]. We conducted GLMM analyses in R, using the “lmer” function in the “lme4” package. Table 2 details all GLMM analyses conducted.

We calculated local tree cover using ArcMap10.0 and a 2010 WorldView II satellite image with 0.5 m-resolution [43]. We performed a supervised classification to define a tree cover layer, and validated the tree cover classification through GPS-based ground-truthing. We used this tree cover layer to calculate tree cover within a 120 m radius about each survey location (this distance avoids overlap of radii between points; see Appendix A).

## Results

We collected 7,724 ichneumonid individuals, and identified 96% (7,399) to species and the rest (4%) to genus (Table 1). Our reference collection of 21 morphospecies comprised 17 species-level identifications, and four morphospecies identified to genus. Of all individuals sampled, 3.5% by abundance (273 individuals) were identified as native to Hawai’i and 97% (265) of these natives were in the genus *Spolas* (we identified these as a single morphospecies, *Spolas* sp.1). The other natives sampled were *Pristomerus hawaiiensis* (six individuals) and *Enicospilus sp.1* (two individuals). Unless otherwise indicated, we pool abundances of the three native species in analyses of native ichneumonids below. We found 18 non-native morphospecies, three of which were numerically dominant – *Agasthenes swezyzii* (3,127 individuals, 41% of total catch), *Vulgichneumon diminutus (*1,381, 18%) and *Woldstedtius flavolineatus* (1,380, 18%). The next most abundant species were an order of magnitude less common.

### Comparison of the Abundance and Species Richness of Ichneumonidae

**Figure 2:**
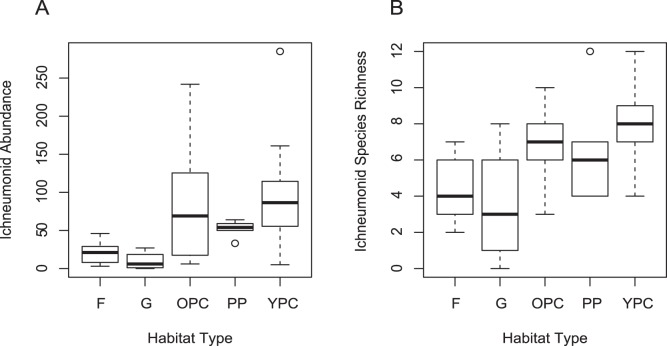
Wasp community characteristics in different habitat types. Panel A represents abundance; panel B represents richness. Habitat types are forest (**F**), grasslands (**G**), old and young planted corridors (**OPC** and **YPC**, respectively), and planted patches (**PP**). Open circles represent outliers.

We found that the richness and abundance of ichneumonid communities were both higher in reforested areas (both patches and corridors) than in grassland (GLMM: z = 5.50; *p*<0.0001 (richness) and z = 6.08; *p*<0.001 (abundance)) and forest (GLMM: z = −3.54; *p*<0.0001 (richness) and z = −4.54; *p*<0.001 (abundance)). Richness values were about twice as great in reforested areas, and abundance values were 5–10 times higher (Fig. 2).

### Comparison of Wasp Communities

Most of the Ichneumonidae collected in all habitat types were non-native to Hawai’i; non-native wasps comprised 100% of wasps sampled in grassland (74 individuals), 97% of wasps sampled in reforested corridors and patches (5,423, of a total of 5,584), and 92% (971 of 1,059) of wasps sampled in forest.

**Figure 3:**
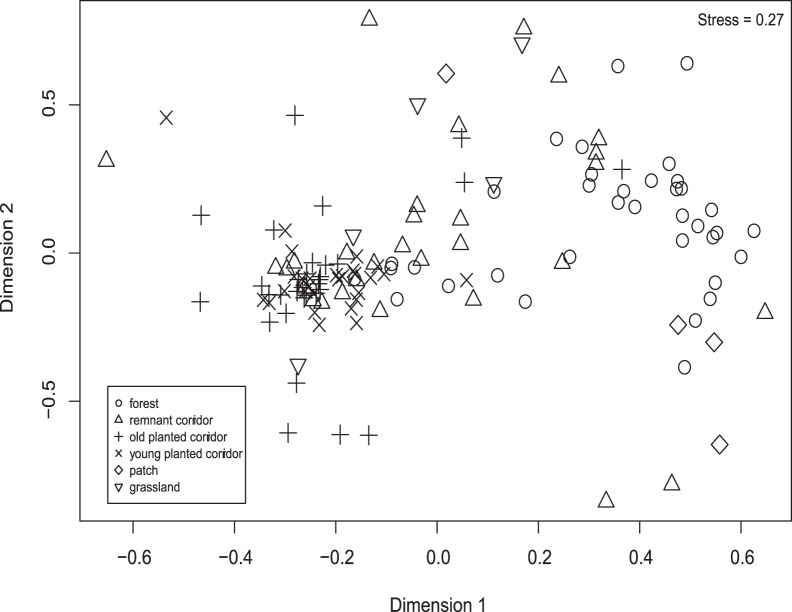
Multi-dimensional scaling plot. Plot demonstrates similarities in ichneumonid community composition in different habitat types.

Differences in the ichneumonid communities existed among all six habitat types (F = 4.17, *p = *0.001, df = 4,24) (Table 3, Fig. 3). We focus our pairwise comparisons on reforested areas, grassland, and forest, since these habitat types are most relevant for restoration. Ichneumonid community composition differed between the grassland and reforested areas (corridors and patches) (F = 8.6, *p = *0.001, df = 1,19). Ichneumonid communities in the native forest were significantly different from those in all planted sites (patches and planted corridors) (F = 13.49, *p = *0.001, df = 1,23). This difference held for comparisons between native forest and planted corridors alone (F = 16.64, *p = *0.001, df = 1,18) and between native forest and patches alone (F = 5.77, *p = *0.003, df = 1,15). We found higher abundances of native ichneumonids in native forest than in all corridors (z = −2.00, *p = *0.045), in forest than in remnant corridors (z = −2.832, *p* = 0.004), and in planted than in remnant corridors (z = 2.48, *p = *0.013). We found no difference in native ichneumonid abundance between native forest and planted corridors (z = −0.913, *p = *0.361), nor between young and old planted corridors (z = −0.890, *p = *0.373).

**Table 3:** Community similarity of all ichneumonids among habitat types of interest in this study.

Analysis	*df*	SS	MS	*F*	R^2^	*p*
**Grassland v. restoration plantings** (old planted corridors, young planted corridors,and patches)	1,19	1.60	1.60	8.6	0.31	0.001
**Grassland v. restored corridors** (old planted corridors and young planted corridors)	1,10	1.85	1.85	23.84	0.70	0.005
**Grassland v. patches**	1,7	1.13	1.13	6.32	0.47	0.007
**All habitats** (native forest, native forest remnant corridors, old planted corridors,young planted corridors, patches, and grassland)	4,24	2.57	0.64	4.17	0.41	0.001
**Native forest v. restoration plantings** (old planted corridors, young plantedcorridors, and patches)	1,23	1.85	1.85	13.49	0.37	0.001
**Native forest v. restored corridors** (old planted corridorsand young planted corridors)	1,18	1.83	1.83	16.64	0.48	0.001
**Native forest v. patches**	1,15	0.80	0.80	5.77	0.28	0.003
**Native forest remnant corridors v. restored forest corridors** (old plantedcorridors and young planted corridors)	1,10	0.41	0.41	2.29	0.19	0.078
**Old planted corridors v. young planted corridors**	1,10	0.16	0.16	1.67	0.22	0.313
**Restored forest corridors** (old planted corridors and young planted corridors)**v. patches**	1,11	0.34	0.34	2.33	0.17	0.130

These PerMANOVA analyses can be viewed as statistical representation of the relationships expressed in the MDS plot (Fig. 3). Higher R^2^ and F values indicate greater dissimilarity. Degrees of freedom are given for the variable and then the residuals.

Ichneumonid communities did not differ by planting configuration (F = 2.33, *p = *0.130, df = 1,11). In addition, communities found in remnant corridors were not significantly different from those in planted corridors (F = 2.29, *p = *0.078, df = 1,10). Nor did ichneumonid communities differ by planting age (F = 1.67, *p = *0.313, df = 1,6).

### Effects of Tree Cover

**Figure 4:**
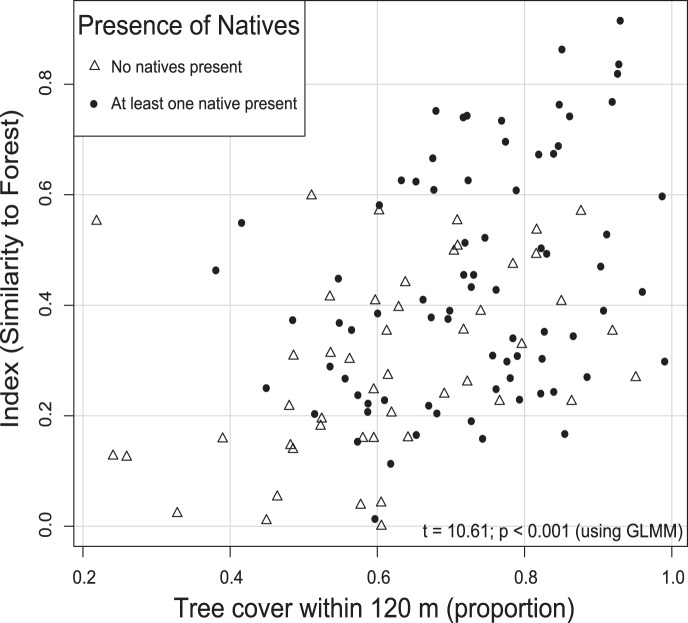
Relationship between similarity-to-forest index and percent tree cover. At points represented by a circle we found no native individuals; at points indicated by a triangle we found at least one native individual. Tree cover is calculated within a circle of 120 m radius surrounding each sampling point in all corridors. Our GLMM analysis found the trend of increasing similarity to forest with increasing tree cover significant.

The community similarity-to-forest index was significantly correlated with tree cover within a 120 m radius of each sampling point (t = 10.61; *p*<0.001; Fig. 4). We found no relationship between native ichneumonid abundance and tree cover (z = 1.33; *p = *0.183). Despite a lack of statistical significance, we observed three general patterns (Fig. 4): (1) when surrounding tree cover within 120 m was higher than 90%, native ichneumonids were present about 90% of the time; (2) native ichneumonids were encountered about 60% of the time when surrounding tree cover was between 38 and 90%; and (3) native ichneumonids were not found when surrounding tree cover was less than 38%.

### Effects of Distance to Native Forest

**Figure 5:**
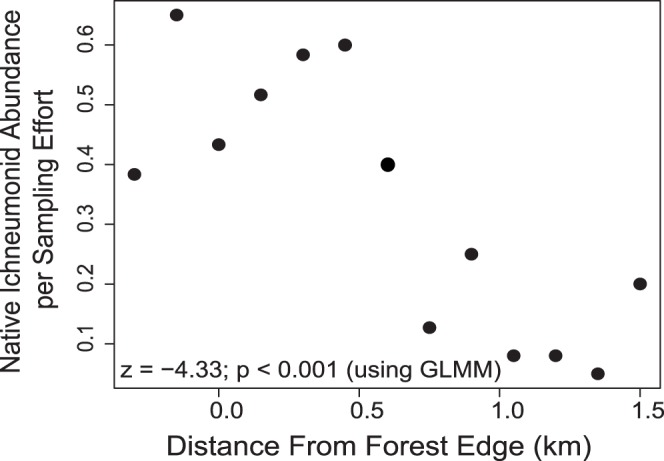
Abundance of native Ichneumonidae with increasing distance from native forest. Our GLMM analysis found the trend of decreasing abundance of native species with increasing distance to forest significant. Values less than zero on the *x*-axis indicate points within the native forest.

We found a positive relationship between native ichneumonid abundance and proximity to forest (Fig. 5, z = −4.33, *p*<0.001). We found no significant relationship between distance to forest and ichneumonid communities, however, presumably because native ichneumonid individuals are so few; the community similarity-to-forest index was not correlated with distance to forest (t = 0.855, *p = *0.393).

### Effects of Non-koa Plant Community Composition

Among understory plant species surveyed (Appendix B), two were significantly correlated with the presence of *Spolas* sp.1 (we used only *Spolas* sp.1 in understory analyses because no other native (morpho)species was sufficiently abundant for analysis). Presence of the native understory tree *Myrsine lessertiana* (kōlea) was positively correlated with presence of *Spolas* sp.1 (z = 2.77; *p = *0.006); conversely, presence of the native fern *Pteridium aquilinum var. decompositum* (bracken fern) was negatively correlated with *Spolas* sp.1 presence (z = −2.31; *p = *0.021).

## Discussion

The Hakalau Forest National Wildlife Refuge restoration effort aims to re-create in former pasturelands a forest that supports the same species that occurred prior to cattle ranching and still occur in adjacent mature forest. HFNWR is particularly concerned with aiding in the recovery of endangered flora and fauna [44]. Although restoring ichneumonid populations is not an explicit management objective for HFNWR, the refuge’s goal of providing habitat for Hawaii’s native species [45] encompasses native ichneumonids. Refuge scientists are also particularly interested in ichneumonid populations because non-native parasitoid wasps may contribute to the decline of bird populations through reduction of important insect prey [25]. Non-native species are an important consideration for most Hawaiian restoration efforts [28], [46]; in HFNWR, particular non-natives species of concern include feral pigs (*Sus scrofa*) [47], several highly invasive plant species, and the approximately 25% of the avian population comprised of non-native birds [48], [49].

### Non-native Species and ‘Novel Ecosystems’

Given that 96.5% of our ichneumonid samples comprised non-native species, at least the arthropod component of our study area could be considered a novel ecosystem [50]. In such novel ecosystems, management approaches that recognize and account for novel species assemblages are needed because complete eradication of non-native species is likely infeasible [51]. While most restoration approaches in Hawai’i are attuned to the roles of non-native species, they often lack important knowledge (for example, of population levels and species interactions) that could guide management decisions.

Ecological restoration could be considered a sub-field of “intervention ecology,” a moniker alluding to both the human agency in restoration and the unlikelihood, in many circumstances, of fully re-creating a pre-existing system [52]. The high proportion of non-native species found in our study begets many questions about restoration trajectories and how human “interventions” impact ecological interactions. In the context of a restoration intervention such as this one, for instance, it is possible that the dominance of non-native species may decline as the ecosystem responds to restoration efforts [53]. If and how interactions between native and non-native species change over time in restoration projects, however, are important questions that warrant further inquiry, because detailed information on ecological interactions is often lacking, as it is in our study system. Our analysis did not include lepidopteran larval rearing studies, an important step in determining ichneumonid impact on ecological communities [26]. Given that HFNWR was created to protect increasingly rare native birds whose diets comprise lepidopteran larvae, and given the overwhelming dominance of non-native Ichneumonidae in this system, such studies are especially important. To add to the complexity, we do not know if the overall abundance of ichneumonids is now higher due to the influx of non-natives. Higher populations would likely negatively impact prey species populations, at least in the short term. Conversely, it is possible that non-native ichneumonids are filling niches left vacant by extirpated or rare native ichneumonid species [54]. Further study of this novel ecosystem would help to illuminate these interactions and impacts.

### Patterns Important for Restoration Efforts

We found significantly higher abundances and greater species diversity of ichneumonids in planted sites than in adjacent grasslands and in the target ecosystem. We found that ichneumonid communities in all planted sites differed from the target system, which is consistent with many past studies of arthropod communities and restoration, for instance in Wyoming [16], Arizona [17], and California [9]. Other past research in agricultural landscapes (as opposed to lands undergoing native habitat restoration) has found greater species richness of bees and wasps in agricultural systems than in protected forest [55]. The consistency of our results with these previous findings leads to questions about the mechanisms underlying higher diversity of arthropod communities in agricultural and restored habitats compared with arthropod communities in native forest.

As in other studies [56] investigation of the relationship between different aspects of ichneumonid community composition and site characteristics yields information pertinent to restoration and management. Although plantings had greater overall (native and non-native) ichneumonid abundance and species richness than native forest, the abundance of native ichneumonids did not differ significantly between plantings and native forest. This is an important distinction that is encouraging for native-focused restoration efforts. We found that restoration of native forest may help to increase the abundance of native Ichneumonidae with respect to non-natives. Specifically, we found native ichneumonids only in native habitat and restored areas (not grasslands), and these individuals were more abundant in areas with higher surrounding tree cover. Further studies are needed to explore the effect of restoration on interactions between native and non-native members of this ecological community.

In corridors, we found a positive relationship between native ichneumonid abundance and proximity to forest. This result, combined with the lack of a difference in native ichneumonid abundance between forests and planted corridors, confirms that the majority of native ichneumonids in corridors are found closer to the forest. The implication of this finding (that native ichneumonids are venturing from the forest into corridors) is an important consideration for restoration, especially in non-native-dominated systems. This result is consistent with past work, which has found that connectivity to target habitat can increase restoration success [57].

We also found that, in corridors, overall tree cover at a relatively fine scale (120 m) is related to the similarity between a given point’s ichneumonid community and the community in the target native forest ecosystem. Research on other highly mobile taxa, such as birds [58], [59], flying beetles [57], butterflies, and moths [60], similarly found that total area of native vegetation is a strong determinant of target species presence in restoration sites.

These two findings, that ichneumonid communities are related to both proximity to native forest and the proportion of tree cover in the surrounding landscape, are consistent with prior work on the distribution of wasps in heterogeneous human-modified landscapes [61] and suggest that landscape context is a critical concern for restoration planning [62]. Our results related to tree cover suggest the importance of trees in particular to ichneumonids. Although we found ichneumonids throughout restoration corridors (up to 1.5 km from native forest), we do not know how far they will fly over non-preferred habitat (in this case, former pastureland) to reach preferred habitat (in this case, trees). Restoration in general should consider that the distance between a restoration site and ‘source’ habitat may be a critical factor for colonization by many taxa, and particularly arthropods [63]. Other factors, however, may outweigh distance, especially for highly mobile arthropods such as butterflies and moths [64], [65]. Each species’ colonization ability will interact with distance between habitats, and for arthropods, as with many other taxa, that interaction will be critical to restoration success [56]. Our results do not address these issues directly, but raise important questions for future research.

That neither native wasp abundance nor overall community composition differ by age of planting is not surprising given ichneumonids’ mobility [23] and the relative maturity of even our youngest sites. This result nevertheless demonstrates that ichneumonids visit restoration plantings within 10 years (this study does not reveal whether the ichneumonids were feeding, reproducing, or passing through the restoration sites). Although a past study found arthropod communities more similar to the target ecosystem in older plantings (6–17 years as opposed to 2–4 years) [56], rapid colonization (in <5 years) is possible even for taxa with relatively low mobility, such as wingless arthropods [66]. It is possible that plantings in our study are in a slower-growth stage of a non-linear transformation process; that is, major changes may have occurred within the first five years (as in [56], [66]), but subsequent changes (e.g., in years 5–20 after restoration) occur more slowly.

Just as age of planting did not seem to impact ichneumonid communities, our results do not suggest that the configuration of planted trees (i.e. patches versus corridors) impacts ichneumonid community composition. This finding is consistent with the scarce past work on arthropods and restoration configurations. Ingham and Samways [67] found that Hymenopterans (wasps, bees, and ants) in South Africa were not restricted to forest fragments and patches; their distributions had no relationship to obvious landscape boundaries.

Native *Spolas* sp.1 were widespread at HFNWR, and their occurrence was positively and negatively correlated with a native sub-canopy tree species (*Myrsine lessertiana*) and native bracken fern (*Pteridium aquilinum var. decompositum*), respectively. Future research could focus on understanding the potential for certain plant species in this system to facilitate the return of particular invertebrate species, for example through provision of native host insects. Previous work has demonstrated this role for plants in other systems. In one site in southwest Australia, for instance, most restored plant species supported distinct assemblages of hemiptera (‘true bugs’) [68]. In a site in the United Kingdom, insect assemblages in native tree plantations with understory restoration plantings were more similar to target habitat than assemblages in native tree plantations lacking understory plantings [69]. In a third site in California, not only plant species composition but also genotype composition affected arthropod community composition [70]. These past findings suggest important considerations for restoration efforts and, combined with our results, inspire questions about plant-arthropod interactions in our study system. For example, does *Spolas* sp.1 parasitize a species hosted by *M. lessertiana*? Conversely, are other plants (in this case *P. aquilinum*, which spontaneously colonized our study area) associated with factors that make an area less hospitable to native Ichneumonidae?

### Limitations

We sampled only in summer months; variations in multiple domains (e.g., floral resource availability; weather) suggest that results could differ with year-round sampling [71], [72]. Inter-annual variation (e.g., droughts or major storm events) could also impact ichneumonid communities [73]. Further, findings might well change with a longer temporal scale than our 16-month period. Additional limitations arise from the spatial arrangement of habitat types. Remnant corridors follow gulches, and thus have different geomorphological characteristics than planted corridors. Young planted corridors are all on the northern edge of the study area and grasslands are nearer the western edge of the study area, while the other habitat types are more evenly distributed. We do not perceive these limitations as great because overall biotic and abiotic conditions in the study area are quite similar.

An additional limitation may stem from the likely impact of edge effects (the variation in species populations at the nexus of two habitat types separated by an abrupt edge [74]). Because planted corridors in our study were approximately 40 m wide, entire corridors could be considered edge habitat [75]. While a number of studies have found edge effects for invertebrates between forest and grassland [76], [77], others have not [64], [78]. Our forest sampling sites that were farthest from an edge were 300 m into the forest; it is possible, and perhaps likely, that edge effects impact these areas as well. One study, for instance, found that edge effects for arthropods (beetles) extended as far as 1 km [79]. Because all corridors were roughly the same width, the likely effect of habitat edges does not jeopardize our results. As restoration proceeds and the corridors widen and eventually meet, these edge effects will be greatly reduced.

### Conclusions

The results of this study can inform restoration action and future restoration research. Although most land stewards do not manage explicitly for ichneumonids, these insects may serve as indicators of various ecological processes [20], [21], [24] and may play critical roles in inter-specific interactions such as parasitism. A better understanding of the effects of time, design (corridor or patch), and landscape context (surrounding tree cover and distance from native, mature forest) on these ecologically important organisms will help landowners, public agencies, and conservation organizations to understand more about the benefits of restoration projects to a broad array of species and will aid efforts to maximize the conservation value of planted forests. Our study suggests that restoration plantings attracted native ichneumonids in numbers similar to native forest, and they also attracted a diversity of non-native wasps. Future restoration interventions may consider implementing restoration plantings in areas with higher surrounding tree cover and biotic connections (e.g., corridors) to native forest, and/or *creating* surrounding tree cover and connections to native forest through restoration plantings, to increase the chance that ichneumonid communities will more closely resemble those of existing native ecosystems. Our findings also reinforce the need for future restoration efforts, especially those in novel ecosystems, to consider the role of non-native species in recently restored ecosystems.

## Supporting Information

Table S1 **All plant species identified during vegetation sampling.**
(DOCX)

File S1 **Elaboration of Methods.**
(DOCX)
