# Supplementary material for: Forest Restoration and Parasitoid Wasp Communities in Montane Hawai’i
Source: PLoS One. 2013 Mar 19;8(3):e59356. doi: 10.1371/journal.pone.0059356 (PMC3601962; doi:10.1371/journal.pone.0059356)
Supplement: Table S1 — All plant species identified during vegetation sampling. (DOCX) [file pone.0059356.s001.docx]

**Table S2. All plant species identified during vegetation sampling.**

All plant species identified during vegetation sampling (scientific and common names). Also indicated is whether the species is Native, Exotic, or its provenance Unknown.

| ***Scientific Name*** | **Common Name**  Hawaiian (English) | **Native, Exotic, or Unknown?** |
| --- | --- | --- |
| *Cheirodendron tryginum* | 'ōlapa | Native |
| *Cibotium glaucum* | hāpu'u (tree fern) | Native |
| *Coprosma montana* | pilo | Native |
| *Cryptomeria japonica* | (tsugi pine) | Exotic |
| *Dicranopteris linearis* | uluhe | Native |
| *Dryopteris sp.* | (none) | Native |
| *Dryopteris wallichiana* | lauhala (shuttlecock fern) | Native |
| *Geranium spp.* | geranium | Exotic |
| *Ilex anamola* | kawau (Hawaiian holly) | Native |
| *Ilex spp.* | (holly) | Exotic |
| *Juncus sp.* | (rush) | Unknown |
| *Metrosideros polymorpha* | 'ōhi'a | Native |
| *Myoporum sandwichense* | naio | Native |
| *Myrsine lessertiana* | kōlea | Native |
| *Ophioglossum sp.* | (none) | Unknown |
| *Passiflora mollisima* | (banana poka) | Exotic |
| *Pteridium aquilinum var. decompositum.* | kilau (bracken fern) | Native |
| *Ranunculus spp.* | (buttercup) | Exotic |
| *Rubus hawaiiensis* | 'ākala (Hawaiian raspberry) | Native |
| *Rubus spp.* | (blackberry) | Exotic |
| *Rumex spp.* | (dock) | Exotic |
| *Sadleria sp.* | 'ama'u (tree fern) | Native |
| *Sophora chrysophylla* | māmane | Native |
| *Leptecophylla tameiameiae* | pūkiawe | Native |
| *Ulex spp.* | gorse | Exotic |
| *Unidentified mistletoe* | (mistletoe) | Unknown |
| *Vaccinium calycinum* | 'ōhelo | Native |
| *Vaccinium reticulatum* | 'ōhelo | Native |
