# Supplementary material for: Forest Restoration and Parasitoid Wasp Communities in Montane Hawai’i
Source: PLoS One. 2013 Mar 19;8(3):e59356. doi: 10.1371/journal.pone.0059356 (PMC3601962; doi:10.1371/journal.pone.0059356)
Supplement: File S1 — Elaboration of Methods. (DOCX) [file pone.0059356.s002.docx]

**S1. Elaboration of Methods**

**Study Site**

We studied differences in old planted corridors and young planted corridors, but grouped planted patches into one analysis category. We made this choice due to the study design and details of the landscape – we had only five patches total, making statistical comparisons between subsets of the patches extremely difficult.

**Statistical Analyses**

**Spatial autocorrelation.** We tested all response variables for spatial autocorrelation using Moran’s I. All response variables exhibited spatial autocorrelation; this was one of the reasons for our use of Generalized Linear Mixed Models (described in detail below in this Appendix).

The values for Moran’s I, and corresponding *p*-values, are in the table below.

| **Variable** | **Moran’s I** | ***p*-value** |
| --- | --- | --- |
| Abundance (all species) | 0.10 | < 0.001 |
| Spp Richness (all species) | 0.09 | < 0.001 |
| Abundance (native species only) | 0.02 | 0.003 |
| Spp Richness (native species only) | 0.06 | < 0.001 |
| Similarity-to-forest index | 0.09 | < 0.001 |

**Table S.1** Moran’s I and *p*-values for response variables.

**Tree Cover Data Analysis.** We calculated the amount of tree cover surrounding each sampling point at radii of 10 meter intervals from 10m to 200m, and then ran Pearson correlation coefficients between tree cover at each radius and the similarity-to-forest index.

We conducted our analyses using tree cover data from within a 120m radius circle of each point for two reasons: (1) in a correlation test between tree cover at multiple radii and the Pearson correlation coefficients peaked at a radius of 120m (*r = 0.44*) and (2) this radius reduces spatial autocorrelation in the tree cover variable, because corridor sampling points were 150m apart.

**PerMANOVA Analysis.**

***Selection of sampling points used in PerMANOVA analyses:*** To avoid pseudoreplication, we pooled temporal replicates and used only one sampling point from each corridor, patch, and grassland. We used the single middle sampling point in each corridor for all but one analysis; the one exception was comparisons involving corridors and grasslands, in which we used the highest sampling point in each corridor because grassland sampling points were at higher elevations in the study area (see Fig. 1). In grasslands and patches we used the center sampling point.

***Confirmations of PerMANOVA results*:** To check our conclusions from all PerMANOVA analyses, we also computed the PerMANOVA using the Jaccard similarity index (in addition to the Chao dissimilarity index); overarching results and conclusions were the same. Also as a confirmation, we repeated the analyses comparing planted corridors to grasslands with all of the non-forest corridor points (not just the highest or middle points). Including all points led to pseudoreplication, however; we did this analysis only to illustrate that the results are not an artifact of characteristics unique to a single point within each corridor. Using PermANOVA to compare all planted sites (all points in Old and Young Planted Corridors, and one point from each Patch), returned the same conclusions as the analysis including only highest-elevation corridor points.

**Details of Generalized Linear Mixed-Effects Models (GLMMs)*.*** Our data were grouped in various ways: a given corridor or patch comprises plantings of the same age; points in each corridor are connected spatially and biotically via contiguous tree cover; remnant corridors are also connected hydrologically because they follow gulches; and we sampled five times at each point. Use of GLMMs for the majority of our analyses allowed us to account for the heterogeneity of our study design and avoid pseudoreplication. Table 2 (in the paper) presents a summary of these analyses in tabular form; we present details in the text below.

To assess differences between grasslands and planted sites in more absolute terms (as opposed to relative relationships in community composition), we used GLMMs with a Poisson error structure to compare the richness and abundance of ichneumonids in planted sites (both patches and corridors) to grassland sites; we excluded forest points and remnant corridors from this analysis because our interest was in whether ichneumonids were found in restoration plantings. The fixed effect in this analysis was habitat type; random effects were specified as point within corridor, patch, or grassland site.

To assess relationships between distance from the edge of mature native forest, tree cover, and our similarity-to-forest index, we used a Linear Mixed-Effects Model with normal error structure. We used only corridor and forest points in this analysis, because the distance measurement has a different meaning in patches, which are not contiguous with the forest. We omitted all forest points 300 m into the forest, because including them would involve comparing each point to itself (since they are the target community in the similarity-to-forest index calculation). We also used points only up to a distance of 1.5 km from the forest, as beyond this distance, we had only one or two points at each distance. Important to note is that, perhaps counter-intuitively, the distance from forest and tree cover terms were not strongly correlated (*r* = -0.196). Despite this fact, however, in our second refinement of this model we dropped distance from forest, as it was far from significance (*p* = 0.393). This model used fixed effects of distance from forest and tree cover; random effects were point nested within corridor (as described in the main article text).

We used a GLMM with Poisson error structure to explore relationships between native ichneumonid abundance, tree cover, and distance from forest. As with the analysis of the similarity-to-forest index, only corridor points up to 1.5 km from forest were used; in this case, however, we included the points 300 m into the forest. Fixed effects were distance from forest and tree cover. Random effects were point nested within corridor.

To investigate the relationships between native ichneumonid abundance and habitat type, we used a GLMM with Poisson error structure. Habitat type was the fixed effect, and random effect was point within corridor or patch.

Finally, to explore relationships between plant community composition and the presence of *Spolas* sp.1, we used a GLMM with a binomial error distribution to compare the presence/absence of *Spolas* sp.1 with the presence/absence of every plant species found near our sampling points. We refined this model based on AIC (stepwise removal of non-significant terms).
